# Ultrafiltration and Fluid Excretion in Echinoids Involves the Axial Organ with Elimination via the Intestine

**DOI:** 10.3390/life15050767

**Published:** 2025-05-10

**Authors:** L. Courtney Smith, Thomas M. Hill

**Affiliations:** 1 Department of Biological Sciences, George Washington University, Suite 6000, Science and Engineering Hall, 800 22nd Street NW, Washington, DC 20052, USA; 2 Department of Microbiology and Immunology, University of Maryland, Suite 380 Health Science Research Facility-I, 685 West Baltimore Street, Baltimore, MD 21201, USA; thomas.hill@som.umaryland.edu

**Keywords:** *Strongylocentrotus purpuratus*, sea urchin, invertebrate, echinoderm, *Ginglymostoma cirratum*, haemal system, podocyte, slit diaphragms, bipinnaria

## Abstract

Many animals display nephridial structures for the ultrafiltration of metabolic waste. However, a nephridial equivalent and an excretory system are not generally recognized for echinoderms. Podocytes are nephridial cells that function in ultrafiltration of body fluids. Limited ultrastructural analyses of echinoderms identify cells with podocyte morphology in the axial organ and in the left coelom of larval sea urchins. Echinoid internal anatomy suggests that the excretory system functions by ultrafiltration in the axial organ, as well as filtrate flow via the water vascular system for excretion through the madreporite; however, these reports are based on morphology. To verify podocytes in the axial organ, orthologues of podocyte-specific genes were evaluated in the sea urchin genome and RNAseq data sets. To verify excretion from the madreporite, fluorescein was used as a tracer for nephridial clearance, and was injected into the main body cavity of sea urchins. Results showed that genes encoding proteins that function in podocytes of vertebrates are expressed specifically in the axial organ of sea urchins, in agreement with orthologue expression in the nurse shark kidney. However, fluorescein clearance from the body cavity shows elimination from the anus rather than the madreporite. This leads to the hypothesis that fluorescein and metabolic waste clearance occur through ultrafiltration by podocytes in the axial organ, but that the filtrate flows into the haemal system and the haemal capillaries in the intestinal walls, from which fluid is transferred to the intestinal lumen for elimination through the anus. Future testing is proposed to evaluate fluorescein filtration from the blastocoel of larvae into the left coelom, and for excretion by small or juvenile echinoids that have undergone tissue clearance to visualize the route of fluorescein flow within the internal anatomy of cleared, intact sea urchins.

## 1. Introduction

**Figure 1:**
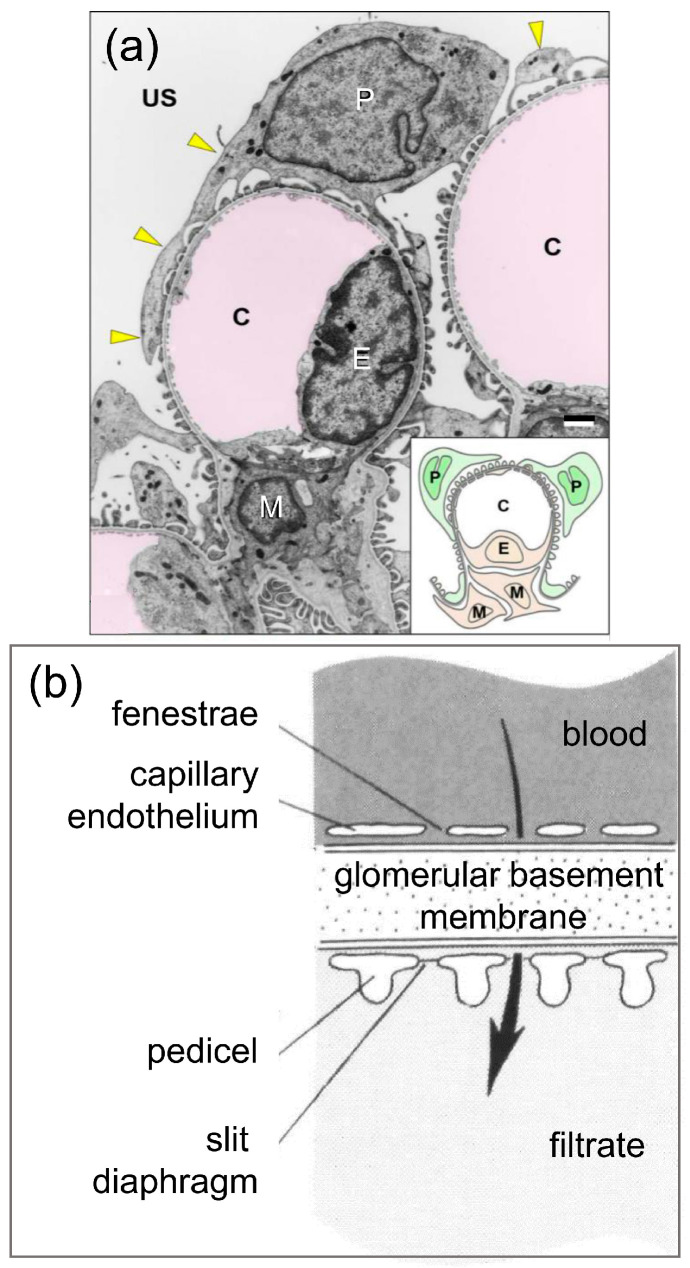
Podocytes and the glomerular structure in vertebrates. (**a**) The glomerular structure in a vertebrate (house musk shrew, *Suncus murinus*) kidney, as imaged in a low-magnification transmission electron micrograph (TEM), shows a podocyte (P) on the ultrafiltrate side (US) of the glomerular basement membrane (GBM) with cellular extensions (primary processes; yellow arrows) that form the pedicels. A capillary endothelial cell (E) is located on the inside of the capillary (C) with fenestrated cellular extensions that cover the GBM. The scale bar in the lower right above the inset indicates 1 µm. A mesangial cell (M) functions to maintain the structure of the glomerulus. A simplified glomerulus is illustrated in the inset. The panel has some minor editing, and is from Ichimura and Sakai [4] in Anatomical Science International, which is open access and is republished under the terms of the Creative Commons Attribution 4.0 International License (http://creativecommons.org/licenses/by/4.0/). (**b**) An illustration of the GBM shows the fenestrated capillary endothelium and the podocyte pedicels with slit diaphragms on the filtrate side. The arrow indicates the direction of filtration flow. The panel includes minor editing to increase the font sizes of some labels. It is republished from Ruppert [7], Evolutionary origin of the vertebrate nephron, Integrative and Comparative Biology, 1994, vol 34, issue 4, pages 542-553, with permission from Oxford University Press.

**Figure 2:**
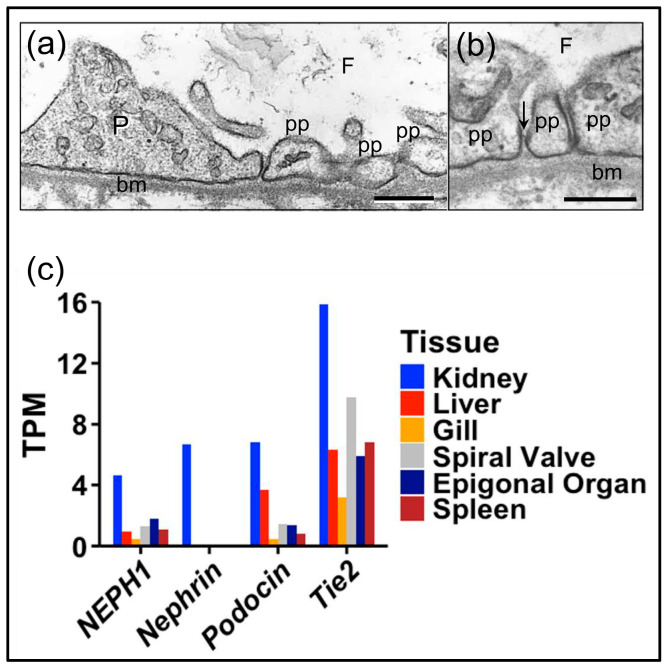
The elasmobranch glomerulus has typical podocyte morphology and expresses podocyte- and glomerulus-specific genes. (**a**,**b**) Transmission electron micrographs (TEMs) of the glomerulus of the little skate (*Leucoraja erinacea*). (**a**) A podocyte (P) with pedicels (pp) that sit on the GBM (bm). The scale bar indicates 0.35 µm. (**b**) A slit diaphragm (arrow) is located between two pedicels (pp) that are positioned on the GMB (bm). The scale bar indicates 0.5 µm. The filtrate side (F) of the glomerulus is indicated in panels (**a**,**b**). See Figure 1 for general vertebrate glomerular microanatomy. Images for panels (**a**,**b**) were provided by Eric R. Lacy. (**c**) Expression of podocyte-specific genes (*podocin*, *nephrin*, *NEPH1*) and a glomerulus-specific gene (*Tie2*) are identified from the transcriptome of the nurse shark (*Ginglymostoma cirratum*). Podocyte and glomerulus marker transcript levels (GenBank BioProject number PRJNA841433) were measured in a suite of tissues harvested from a healthy, juvenile, wild-caught nurse shark, and are mapped onto the transcriptome. Transcript levels in each tissue were calculated using salmon (ver 0.13.1) and read counts were normalized to transcript per million (TPM).

The kidney glomerulus is the site of ultrafiltration in vertebrates. Its structure includes blood capillaries lined with glomerular endothelial cells that cover the glomerular basement membrane (GBM) with fenestrated extensions through which fluid can pass (Figure 1) [1]. Podocytes are located on the filtrate side of the GBM, as well as with cellular extensions that support pedicels, or foot processes, that sit on the GBM. The pedicels generate and maintain slit diaphragms between them that are essential for ultrafiltration [1,2]. Blood pressure drives renal function and the formation of the ultrafiltrate from the movement of serum fluid through the endothelial fenestrations, across the GBM, between the pedicels, and past the slit diaphragms. The glomerular structure in the vertebrates and the presence of podocytes is generally similar, including elasmobranchs and cyclostomes (Figure 2a,b) ([3,4,5] reviewed in [6]).

**Figure 3:**
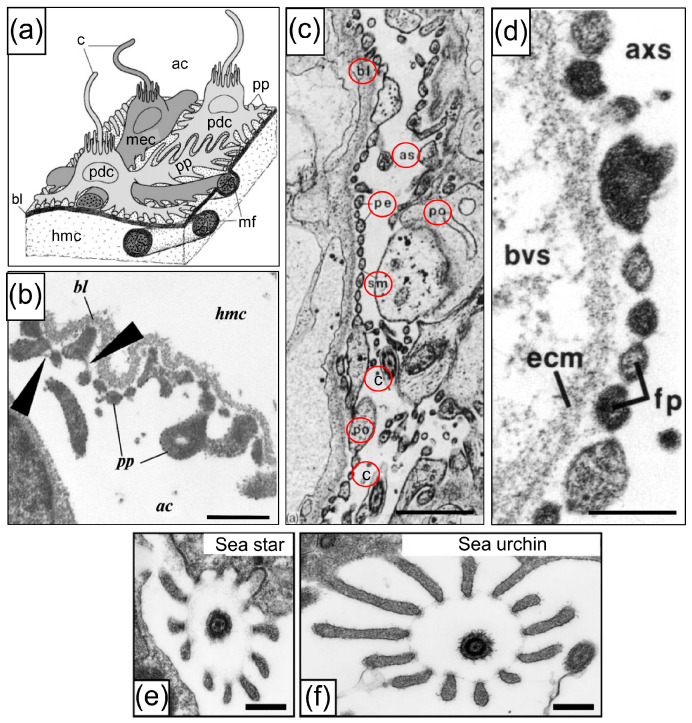
Podocytes in echinoderms are present in the larval left coelom and the adult axial organ. (**a**) A schematic diagram of the ultrafiltration site in the axial organ of an asteroid. The podocytes (pdc) and myoepithelial cells (mec) are located on the basement membrane (basal lamina, bl) of the axial organ. Each cell has a single cilium (c) that is surrounded by a collar of microvilli. The hemocoel (hmc) is located below the bl and the axial coelom (ac) positioned above. (**b**) A TEM of the axial organ of the sea star, *Asterias amurensis*, shows the basal lamina (bl), on which are located the pedicels of podocytes (pp) with slit diaphragms (large arrowheads). The axial coelom (ac) is oriented below the bl, and the haemocoel (hmc) is above. The scale bar indicates 0.5 µm. Panels (**a**,**b**) are from Ezhova and Malakhov [15], Doklady Biological Sciences (available online: https://link.springer.com/journal/10630), and are republished with permission. (**c**) A TEM of the axial organ of an adult sea urchin, *Eucidaris*, shows podocytes (po) with pedicels (pe) and slit diaphragms (slit membranes, sm) along the bl and facing the axial coelom (axial sinus, as). Cross-sections of cilia (c) are evident in the axial coelom. Labels are circled for ease in finding them. The scale bar indicates 1 µm. This panel is from Welsch and Rehkämper [24], ©1987 The Zoological Society of London, and is reproduced with permission from Jon Wiley and Sons. (**d**) A TEM of the axial organ of a juvenile sea star, *Asterias*, shows podocyte pedicels (foot process, fp) on the basement membrane (extracellular matrix, ecm). The axial organ (bvs) is to the left and the axial coelom (axial sinus, axs) is to the right. The scale bar indicates 0.25 µm. This panel is from Ruppert and Balser [25], which is republished with permission. (**e**,**f**) TEM cross-sections of a cilium with the collar of microvilli on podocytes in a sea star and the sea urchin, *Hemicentrotus pulcherrimus*. The scale bars indicate 0.2 µm. Panels (**e**,**f**) are from Ichimura and Sakai [4] in Anatomical Science International, which is open access, and are republished under the terms of the Creative Commons Attribution 4.0 International License (http://creativecommons.org/licenses/by/4.0/).

Animal phyla have been characterized based on the presence of discrete excretory organs. The nephrozoan clade [8,9] includes the bilaterian phyla within the protostomes and deuterostomes, for which most animals have identifiable excretory organs in both larvae and adults (e.g., [10,11]). Other metazoans, including the Porifera, Cnidaria, Ctenophora, and Xenacoelomorpha, have no identified or specific excretory organs. This separation of taxa is supported by genes encoding proteins that function specifically in excretory tissues in nephrozoans, but that are expressed in a broad range of tissues in a xenocoelomorph and a cnidarian, including the epidermis and the gut [12]. Nephridia are filtering units within excretory organs and differ in structure and complexity among the nephrozoa, showing different mechanisms to generate the driving force for filtration. Protonephridia are simpler systems with one or more terminal cells that filter fluids based on flow from ciliary activity. The fluid moves across the basement membrane of a terminal cell, past clefts in pedicel-like structures and across slit diaphragms to a ciliated canal cell that connected to the exterior surface of the animal by a pore [13]. Metanephridial systems are more complex and function based on pressure differences between two apposed coelomic spaces. The pressure moves fluid across the basement membrane, past podocytes with pedicels, and across the slit diaphragms, to form the ultrafiltrate in a second coelomic space [14]. Podocytes typically have a single cilium that is surrounded by a collar of microvilli, they may have basal myofilaments, and may be associated with myoepithelial cells that are also ciliated (Figure 3a) [15,16]. Nephridial systems are present in simpler to complex structures that generally correlate with evolutionary time [7,10,17,18] and are replicated in the ontogeny of many nephrozoans with larval protonephridia and adult metanephridia [13,19]. The morphology of podocytes has been used to identify the location and the structure of excretory systems in a wide range of nephrozoan invertebrates, including molluscs [20], gastrotrichs [21], crustaceans [22], annelids [23], and nemertians [18,19]. The morphology has been verified by orthologous transcription factors that are expressed exclusively in podocytes and kidneys [10].

Nephridial structures are present in most of the invertebrate deuterostome phyla. The hemichordate acorn worm, *Saccoglossus*, has a glomerulus-like nephridium in the proboscis that is composed of capillaries of the circulatory system and podocytes that sit on the basement membrane on the filtrate side in the proboscis coelom [16]. Pressure from the heart moves fluid across the basement membrane of the capillaries and past the processes of the podocytes with pedicels and slit diaphragms and into the coelom of the proboscis. The podocytes are myoepithelial cells with a single cilium and their contractions may augment pressure from heart contractions to drive filtration. The ultrafiltrate in the proboscis coelom flows along the coelomoduct to the exterior through an opening in the neck [16]. Similarly, a cephalochordate, the larval amphioxus, *Branchiostoma*, has a single nephridial structure called Hatschek’s nephridium, located in the head of the animal with a nephridiopore that opens into the pharynx [26]. The epithelial lining of the nephridial lumen is composed of filtration cells that sit on the basement membrane and are structurally consistent with podocytes [6,27]. They have extensions along the basement membrane with pedicels, slit diaphragms, and a single cilium surrounded by microvilli. The nephridium filters the haemal fluid and moves it into the lumen of the nephridium and out the nephridiopore. Adult *Branchiostoma* retain the anterior Hatschek’s nephridium and have multiple branchial nephridia positioned along the trunk associated with each of the pharyngeal slits [28]. They have a simpler structure that is intermediate between proto- and metanephridia. The filtrate formed by the branchial nephridia is released into the atrium (reviewed in [29]).

Unlike the other nephrozoans, the urochordates do not have an excretory organ with a tubular connection to the exterior. This may be the outcome of specialization and reduction of this system over evolution, such that similarities to nephridial systems have been lost [29]. Consequently, a nephridial system in the urochordates has been assumed to be absent [30]. However, tunicates either excrete urea or they store nitrogenous waste, although this varies among species [31,32]. There are two types of renal systems among different groups of urochordates, which are as follows: arenal and renal [33]. The mechanisms of excretion by the arenal urochordates are mediated in part by specialized, migratory blood cells called nephrocytes that store nitrogenous wastes. They are identified by their excretory granules called concretions and are visualized as birefringent cells located along the intestine, in the mantle, and in the subendostylus [32,34]. The renal type of excretory system in some of the urochordates is a large sac in the body wall or mantle located near the pericardium at the base of solitary tunicates [33]. It is ductless sac lined by cells that take up waste from the body fluid and concentrate it into concretions. The fluid stored in the sac is yellowish urine-like fluid, and solid brown, birefringent concretions are released from the cells and accumulate in the sac over the life of the animal [33]. In some species, there are several renal sacs associated with the intestine.

**Figure 4:**
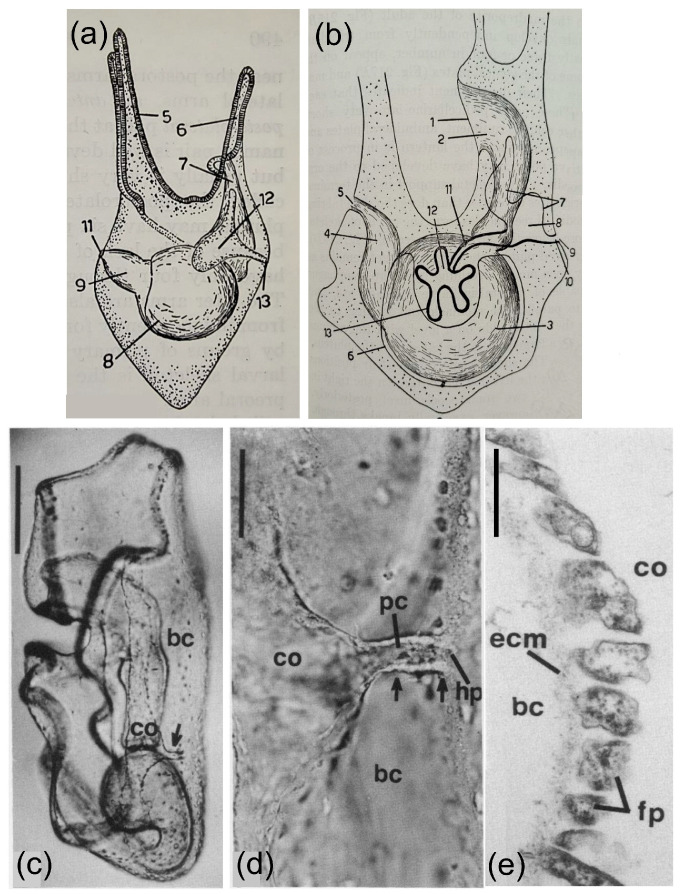
The structure of the larval hydropore system is consistent with metanephridial function. (**a**) The four-arm pluteus larva of the sea urchin, *Psammechinus miliaris*, shows the location of the left coelom or hydrocoel (12) and the water canal that connects to the hydropore (13). Other regions of the larvae include arms (5, 6), esophagus (7) that is located behind the left coelom, stomach (8), intestine (9), and anus (11). This panel is from MacBride [39] with modifications by Hyman [40]. (**b**) A drawing of an older pluteus larva of *P. miliaris*, showing the adult rudiment (12) with primary podia (13), stone canal (11) that connects the rudiment to the left coelom or axocoel (7), the ampulla (8), water or pore canal (10), and hydropore (9). Other structures include the mouth (1), esophagus (2), stomach (3), intestine (4), and anus (5). This panel is from Bury [38], as modified by Hyman [40]. (**c**) The hydropore canal complex of the bipinnaria larva of the sea star, *Asterias forbesi*, is composed of the pore canal (arrow) and left coelom (co). The blastocoel (bc) is also indicated. The scale bar in the upper left indicates 200 µm. (**d**) A higher magnification of the hydropore canal complex in the bipinnaria larva shows the pore canal (pc, arrows) connecting the left coelom (co) to the hydropore (hp) through the blastocoel (bc). The scale bar in the upper left indicates 50 µm. (**e**) A TEM of the bipinnaria larva of *A. forbesi* shows the pedicels or foot processes (fp) of podocytes on the inside surface of the GBM (extracellular matrix, ecm) that surrounds the left coelom (co). The blastocoel (bc) is oriented to the left. The scale bar in the upper left indicates 0.5 µm. Panels (**c**–**e**) are from Ruppert and Balser [25] in the Biological Bulletin, and are republished with permission from the University of Chicago Press.

In contrast to other deuterostomes, echinoderms are not generally described as having an excretory system for the removal of metabolic waste products, even though it is unclear how they survive without one. It is noteworthy that little information on excretion in echinoderms has been included in textbooks on comparative anatomy or physiology. However, there are limited reports in the scientific literature that provide information on excretory systems in echinoderms, and these are generally based on cells with podocyte morphology that are associated with basement membranes. In larval echinoderms, the left coelom has similarities to a rudimentary metanephridial system [25,35,36] based on the structure of the left coelom (or hydroenterocoel) in the four-arm larva of the sea urchin, *Psammechinus milliaris* [36,37], in agreement with Bury [38], MacBride [39], and as reviewed by Hyman [40]. A pore canal connects the left coelom to the hydropore that opens to the exterior on the dorsal side of the larva (Figure 4a–d). Cells that line the basement membrane on the inner side of the left coelom and the water canal have a podocyte-like structure, including pedicels with slit diaphragms that sit on the basement membrane (Figure 4e). This structure is consistent with filtering fluid from the blastocoel through the basement membrane, past the pedicels and slit diaphragms, and into the left coelom. The cells lining the hydropore canal have a single cilium [41] that may function to establish fluid flow out of the left coelom, through the pore canal and the hydropore [25]. Because the left coelom does not collapse as a result of the outflow, this suggests that fluid continues to be filtered as it enters the left coelom from the blastocoel. These results suggest that echinoderm larvae have a metanephridial system that filters blastocoelar fluid. They also have a similar structure and function as the filtering nephridial systems in hemichordates and cephalochordates.

**Figure 5:**
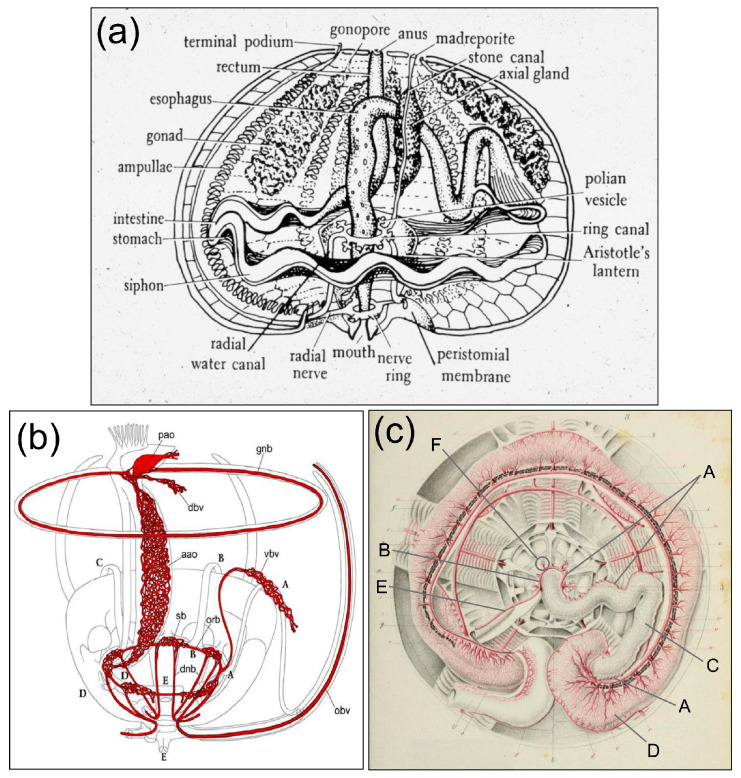
Sea urchin internal anatomy and the haemal system. (**a**) The internal anatomy of an adult echinoid shows the location of the axial organ on the stone canal and its orientation to the esophagus and the rectum. This panel is from Petrukevitch [54] and has been modified by others. (**b**) The haemal system (red) of an adult echinoid shows the axial organ (aao), its connection to the haemal ring (orb) that is located at the top of Aristotle’s Lantern. The ventral haemal vessel (vbv) arises from the haemal ring and branches into the intestinal walls, which are not included in the figure, in the ventral region of the sea urchin. The contractile vesicle (pericardium, pao) is connected to the axial organ at the dorsal end. The dorsal haemal vessel (dbv) arises from the dorsal end of the axial organ and branches into the upper intestinal walls that are not included in the figure. The remaining labels in the panel are not relevant to this study. This panel is from Ezhova et al. [53], which is open access and is republished with permission. (**c**) The haemal system (red) in the sea urchin, *Echinus*, shows the esophagus and the ventral (oral) level or first loop of the intestine with the associated haemal capillaries. The ventral haemal vessel (A) arises from the ventral haemal ring (B), runs along the esophagus (C) to the ventral intestine, (D) and branches into capillaries in the intestinal walls. The haemal capillaries are indicated as thinly branching red lines on the intestine. The connection between the axial organ, which is not included in the figure, and the haemal ring is indicated (E), as are Tiedemann’s bodies (F) on the ventral haemal ring, of which one is circled. The dorsal haemal system for the dorsal intestine is not included in the figure. Note that the Polian vesicles in panel (**a**) are not the same as the Tidemann’s bodies in panel (**c**). The remaining labels are not relevant to this study. This panel is from Perrier [55], with labels according to Hyman [40], of which those with relevance to this study have been revised to letters.

Structures in adult echinoderms that may be possible sites of a metanephridial filtering function are suggested based on the microanatomy of the axial organ [14,24,25,35,42,43]. Confusion regarding the axial organ has resulted in various proposed functions, such as a heart [44,45,46] or contractile vessel [47], a site of coelomocyte proliferation ([48] and verified by Golconda et al. [49]) and/or cell degradation [50], or a secretory gland [45,47]. Despite this, it is feasible that the axial organ may carry out some of these functions simultaneously. It is positioned within the axial coelom and is bound to the stone canal in the central axis of the spheroid body of adult sea urchins. The stone canal is attached to the madreporite on the dorsal, or aboral surface of sea urchins, and to the ring canal of the water vascular system that runs around the esophagus at the top of Aristotle’s Lantern on the oral side (Figure 5a) [51,52]. The pulsating or contractile vessel within the pericardial coelom or dorsal sac is located between the axial organ and the madreporite on the aboral side of the animal and undergoes show contractions that have been the basis for the proposed heart function [45,53]. Furthermore, the myoepithelial cells in the axial organ [15,16,44,45] may also be the basis for contractions that are noted in freshly dissected axial organs (LCS; personal observations). Because ultrafiltration in metanephridia and glomeruli function based on pressure differences across a basement membrane, contractions of the contractile vesicle and the myoepithial cells in the axial organ may function to provide this pressure. In addition to the water vascular system, the axial organ is also connected to the haemal system [40,47,52,53], and is associated with the perivisceral coelom, which is the main body cavity and contains coelomic fluid.

The microanatomy of the axial organ in several classes of echinoderms shows cells that are consistent with podocyte morphology, including pedicels and slit diaphragms, that are located on the axial coelom side of the basement membrane that surrounds the axial organ (Figure 3c–d) [7,15,24,25,51,53,56,57]. Furthermore, a characteristic feature of podocytes in echinoderms is a single cilium that may function in fluid movement and is surrounded by a collar of microvilli with morphological similarity to Poriferan choanocytes [58]. The cytological structure of the podocytes in the axial organ of adults (Figure 3a,e,f) [15,36,59] is similar to the cells that line the left coelom and the water or pore canal in larvae [41]. The anatomy of the axial organ in echinoids shows that it is connected to the stone canal and the ampulla of the madreporite on the aboral side of sea urchins [45,51,53]. This anatomy has been used to infer the route of excretory fluid flow from the axial organ to the exterior by way of the madreporite. The podocyte structure and the proposed excretory pathway suggest that echinoderms have a functional excretory system that is consistent with metanephridial functions in other invertebrates. However, the functions of the podocyte-like cells in the axial organ have not been verified with alternative approaches to demonstrate that the route of excretion is from the axial organ to the madreporite ampulla and through the pores of the madreporite to the exterior. To verify the anatomical predictions of podocyte-like cell function in the axial organ, genes with kidney-specific expression in vertebrates were used to identify orthologues in the sea urchin, *S. purpuratus*. The route of excretion from the axial organ to the exterior via the madreporite was evaluated based on the clearance of fluorescein injected into the coelomic fluid. Our findings indicate that the axial organ shows increased expression of podocyte-specific genes relative to other adult tissues, suggesting filtration function, in agreement with orthologous gene expression in shark kidneys. However, the route of fluorescein excretion does not proceed through the madreporite, but is excreted from the anus. This suggests that the predicted excretory route from the axial organ to the madreporite, which is based on anatomy, needs to be reconsidered. Here, we propose a hypothesis for an alternative route for excretion via the haemal system that connects the fluid flow from the axial organ to the intestine. 

## 2. Materials and Methods

### 2.1. Transcript Analyses

Proteins encoded by genes in the genome of the California purple sea urchin *Strongylocentrotus purpuratus* (ver 5.0; accessed April and October 2024) were identified using annotated gene name searches and by BLASTp with podocyte-specific protein sequences from vertebrates. Transcripts from podocyte-specific genes in adult tissues and larvae from the sea urchin were evaluated based on an RNAseq data set that included transcripts per tissue or developmental time point, and were used to infer gene expression, as reported by Tu et al. [60]. The RNAseq data are cited as WHL.22 sequences and can be searched using the genome browser for *S. purpuratus* on Echinobase.org.

Podocyte and glomerulus marker transcript levels (GenBank BioProject number PRJNA841433) were measured in a suite of tissues from a healthy, juvenile, wild-caught nurse shark, *Ginglymostoma cirratum*, and mapped onto the transcriptome [61]. Transcript levels in each tissue were calculated using salmon (ver 0.13.1) [62] and read counts were normalized to transcript per million (TPM).

### 2.2. Sea Urchin Acquisition and Care

Adult sea urchins (*S. purpuratus*) were collected (December 2016) from the shallow waters near Santa Barbara, CA, USA, by scuba diving and held in the flow-through sea water system at the University of California at Santa Barbara. Sea urchins of unknown sex and without signs of spotting disease, drooping spines, or other indicators of disease were used in the study. They were fed freshly collected kelp fronds (*Macrocystis pyrifera*) once a week.

### 2.3. Fluorescein Injection

Optimal fluorescein concentrations for ophthalmological angiography in humans have been reported as 15–30 mg/kg or 5–10 mL of 0.01–0.02% fluorescein solution injected intravenously [63,64]. This resulted in a final concentration in blood for several vertebrate species that was much lower than LD50 data. Therefore, injections of fluorescein into sea urchins were based on the internal volume of *S. purpuratus*, which was calculated from the dimensions of an oblate spheroid with a correction factor for the variability in the shape among individual sea urchins [65].

((test width (cm) × test height (cm))/2) × 4.1888 = internal body volume (mL)

A solution of sodium fluorescein (25 mg/mL; Sigma-Aldrich, St. Louis, MO, USA) in artificial coelomic fluid (aCF, 10 mM CaCl_2_, 14 mM KCl, 50 mM MgCl_2_, 398 mM NaCl, 1.7 mM Na_2_HCO_3_, 25 mM Na_2_SO_4_ [66]) was injected into sea urchins through the peristomial membrane and into the coelomic fluid in the perivisceral coelom. Injected volumes (≤150 µL) were based on the following equation.

(body volume (mL) × 10^3^)/250 = µL of fluorescein solution

This resulted in a final concentration of fluorescein in the sea urchins of about 100 µg/mL. Sea urchins were returned immediately to a glass aquarium with flow-through sea water at the ambient temperature of the near-shore Pacific Ocean of 15–16.5 °C. Sea urchins were also held temporarily in glass culture dishes (11.5 or 21 cm diameter) filled with ambient sea water. All sea urchins (n = 3) survived the needle puncture wounds without adverse effects. Sea urchins were observed for the release of fluorescein, and imaging was carried out with digital cameras under UV light at 365 nm, beginning a few minutes post-injection (mpi), and they were monitored for 10 days post-injection. One sea urchin was also imaged in sunlight at 22–23 h post-injection (hpi).

## 3. Results

### 3.1. Podocyte-Specific Genes Are Expressed in the Axial Organ of Sea Urchins in Agreement with Expression in the Glomeruli of Sharks

The morphological basis for podocyte function with metanephridial filtration in the axial organ of echinoderms requires supporting molecular data. Several proteins essential for the structure and function of slit diaphragms include podocin, nephrin, and nephrin/podocin-interacting protein (NEPH1) ([67,68,69,70], reviewed in [2]). Nephrin and NEPH1 are proteins with transmembrane regions and extracellular domains that extend into extracellular space and are essential for slit diaphragm structure [67,71,72,73]. Podocin is a cytoplasmic protein [74] associated with the plasma membrane and it binds to the cytoplasmic domains of both nephrin and NEPH1 [67]. These molecular complexes link the slit diaphragm to the cytoskeleton in the podocyte and activate cell signaling to maintain or repair the structure of the slit diaphragm for ultrafiltration ([67], reviewed in [1,70]). Homeobox transcription factors encoded by *six1* and *six2* are expressed in mammalian kidneys and function in renal development and maintenance of stem cells ([75,76], reviewed in [77]). Human Tie 2, an angiopoietin receptor, functions in glomerular nephrogenesis and modulates the endothelial cell population in the glomerulus [78,79]. To lend support for podocytes with slit diaphragms in echinoderms, vertebrate orthologues with podocyte-specific function and expression were used to search the proteins encoded in the genome (ver 5.0) and transcriptomes of the sea urchin, *S. purpuratus*. Searches with the sequences for human podocin, nephrin, and NEPH1 resulted in several matches to genes encoding nephrin and Kin of IRRE (*KIRRE*)-like protein 3, which is another name from NEPH1 (Table 1) [67]. Searches with the *Danio* podocin protein sequence and a search using the gene name ‘podocin’ both failed to identify annotated genes in the sea urchin genome. Searches with the gene name *six* identified a sea urchin orthologue, *six1/2*, in the genome. The sea urchin orthologue of vertebrate, *Tie2*, *SpTie1/2*, encodes a receptor tyrosine kinase [80,81] and was annotated in the first build of the *S. purpuratus* genome (ver 1.0) (Table 1) [82]. Although a podocin orthologue was not identified, these results suggested that podocyte-like cells may be present in sea urchins, which is in agreement with cytology for both larvae and adults. 

The genes encoding proteins with a putative podocyte function were also evaluated based on elevated expression patterns in the axial organ compared to other adult tissues, in addition to expression in larvae and the developmental stages of embryos. The RNAseq data set of Tu et al. [60] was interrogated, and it is available as WHL.22 sequences on the genome browser for *S. purpuratus* on Echinobase.org [83]. The results indicated that expression for both *SpNephrin* and *SpKIRRE* (*NEPH1*) were elevated in the axial organ compared to other adult tissues, including developmental stages (Table 1). *SpNephrin* and *SpNEPH1* were expressed in sea urchin larvae at a time point when the left coelom has been proposed to have a metanephridial function. The echinoid orthologue, *six1/2*, is also expressed specifically in the left coelom, the hydropore canal, and the hydropore in developing sea urchins 72 h post-fertilization [41]. The *six1/2* transcript showed elevated gene expression in the axial organ among other adult tissues and in juveniles (Table 1). *SpTie1/2* is expressed in embryos starting at early gastrulation, which corresponds with putative filtering by the left coelom, and expression in adult *S. purpuratus* is elevated in the axial organ and coelomocytes (Table 1), as reported previously [80,81]. The results for podocyte-specific gene expression were consistent with cytology showing podocyte-like cells in the axial organ and the left coelom of larvae, and suggested a possible metanephridial filtering function in both adults and larvae. This was also consistent with the expression of *Nephrin*, *NEPH1*, *SIX1*, and *SIX2* in excretory organs of vertebrates and many invertebrate nephrozoans [10].

The possible metanephridial functions and gene expression of sea urchin podocytes may be different for podocytes in terrestrial or fresh water vertebrates because echinoderms are marine osmoconformers [84]. Consequently, podocyte-specific gene expression in sea urchins was compared to gene expression in elasmobranchs, sharks and rays, which are also marine osmoconformers [85]. This avoided possible evolutionary changes in glomerular ultrafiltration functions and gene expression between marine vs. terrestrial and fresh water animals [17]. Although renal function in elasmobranchs is mediated through urea retention via an elaborate kidney tubule counter current system [86], the glomerular structure of elasmobranchs is consistent with the structure in other vertebrates [5]. The glomerulus in the little skate, *Leucoraja erinacea*, has endothelial cells that line the capillaries, and podocytes with pedicels and slit diaphragms that are positioned on the filtrate side of the GBM (Figure 2a,b). To verify podocyte-specific gene expression in an elasmobranch, transcripts encoding nephrin, podocin, NEPH1, Tie2, six1, and six2 were evaluated in the transcriptome of the nurse shark (*Ginglymostoma cirratum*) (GenBank BioProject number PRJNA841433, accessed on 10 April 2024) [61]. Results showed that podocyte-specific gene expression was elevated in kidneys compared to other shark organs (Figure 2c; Table 2). Orthologues of the *six* genes were not identified, perhaps based on the failure to annotate these transcripts in the nurse shark database. These results indicated that osmoconformers, such as the nurse shark and the sea urchin, expressed podocyte-specific genes in their respective metanephridial and glomerular filtering tissues, in agreement with other vertebrates, of which many are osmoregulators. Overall, our findings indicated that whether or not an animal is an osmoconformer, this did not alter the gene expression or functions of podocytes.

### 3.2. Fluorescein Excretion in S. purpuratus Occurs Through the Intestine and Anus Rather than the Madreporite or Gills

**Figure 6:**
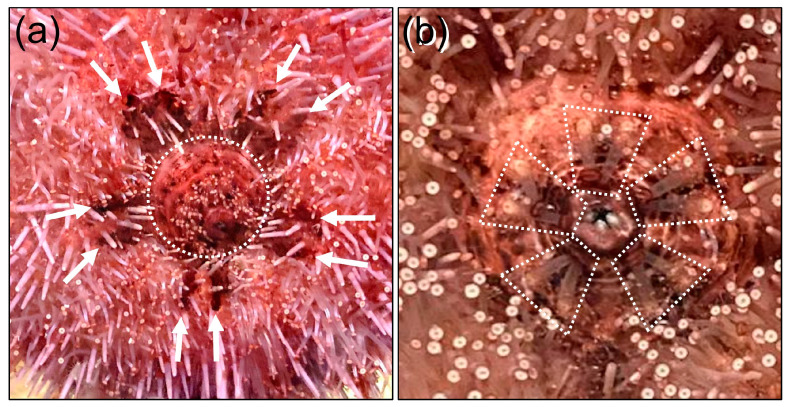
Gills and buccal podia surround the mouth on echinoids. (**a**) Five pairs of gills are present at the edges of the peristomial membrane (dotted circle) and appear as dark patches (arrows) among the spines and tube feet. The diameter of the peristomial membrane is 19.1 ± 0.92 mm, which covers Aristotle’s Lantern (mouth parts) and extends to the gills in adult sea urchins. (**b**) Five pairs of buccal podia (outlined by dotted lines) surround the mouth on the peristomial membrane. Each podium is extended and lying on the peristomial membrane with the suction cups oriented away from the mouth. These images were provided by Julia S. Nadler.

The anatomy of the axial complex in all classes of adult echinoderms, which has been reconstructed from sectioned tissues and magnetic resonance imaging, indicates that the axial coelom is connected directly to the ampulla of the madreporite that is positioned just below and adjacent to the madreporite (Figure 5a,b) [15,16,51,52,53,87,88]. Based on this proximity, it predicts that the ultrafiltrate from the axial coelom moves to the ampulla and is excreted through the ciliated pores in the madreporite. This route of excretion is supported by the metamorphosis of the sea urchin, *Psammechinus miliaris*, in which the larval metanephridial system of the left coelom, water or pore canal, and hydropore, become the axial complex, stone canal, and madreporite, respectively, in the juvenile and adult (Figure 4b), in agreement with the metamorphosis of species in other classes of echinoderms [7,25,35,53,89]. However, the route of an echinoderm excretory system that is based on morphology has not been tested on live animals. Consequently, the metanephridial function of the axial organ and excretion by the madreporite in adult sea urchins were evaluated using a tracer molecule. Fluorescein was chosen because when it is injected into vertebrate blood vessels—typically to evaluate blood flow—it is cleared by ultrafiltration in the glomeruli of the kidneys and appears in the urine [63,64,90,91]. Fluorescein was injected into the perivisceral coelom of adult sea urchins, which contains coelomic fluid that can be viewed as the equivalent of blood, to verify clearance and excretion through the madreporite. In addition to the madreporite, sea urchin gills, which are located at the outer edges of the peristomial membrane (Figure 6a), have also been speculated to function in excretion or respiration [45,92], and were a second possible site of fluorescein excretion.

**Figure 7:**
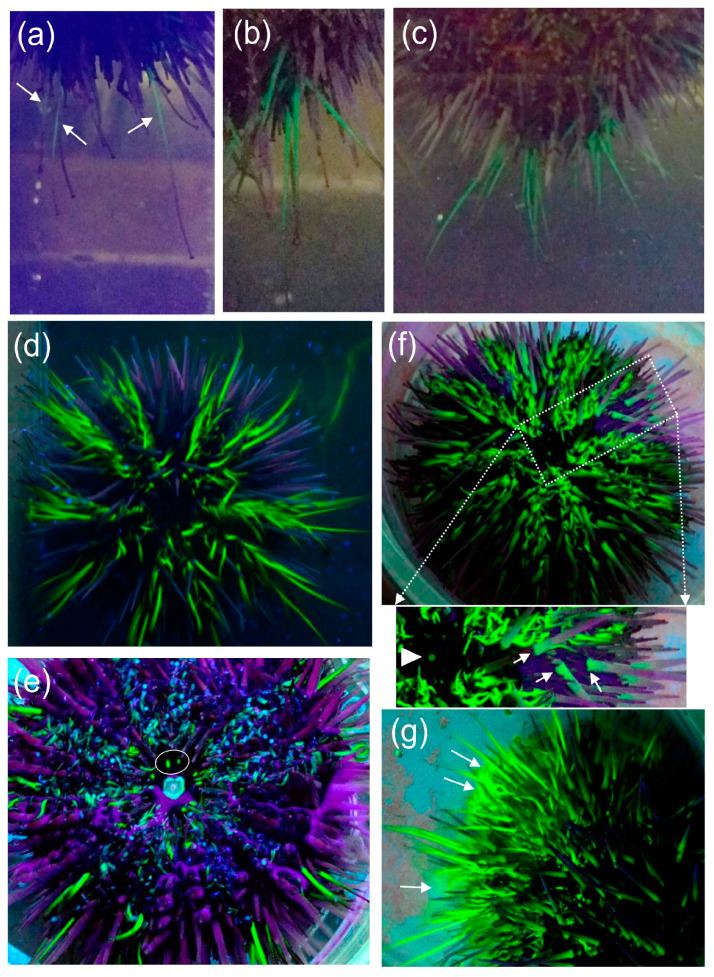
Tube feet and buccal podia become fluorescent after fluorescein injection into the peri-visceral coelom. (**a**) The first evidence of fluorescein in an injected sea urchin is the appearance in a few tube feet (arrows) at 12 min post-injection (mpi). (**b**,**c**) More tube feet become fluorescent at 16 to 23 mpi. (**d**) The dorsal side of a sea urchin shows that all tube feet are fluorescent at 90 mpi. (**e**) The ventral side of a sea urchin in a culture dish at 150 mpi shows that the buccal podia (two are circled) and the edges of the mouth are fluorescent. (**f**) The dorsal side of a sea urchin at 21 h 30 mpi shows that the tube feet remain fluorescent. The insert shows that fluorescence is also evident in the base of the spines (arrows) and at the edge of the anus (arrowhead). (**g**) At 21 h 30 mpi, fluorescein (arrows) appears in the water on the dorsal side of a sea urchin as it turns over in a culture dish. Images in panels (**d**–**g**) were provided by Lee C. Heiman.

**Figure 8:**
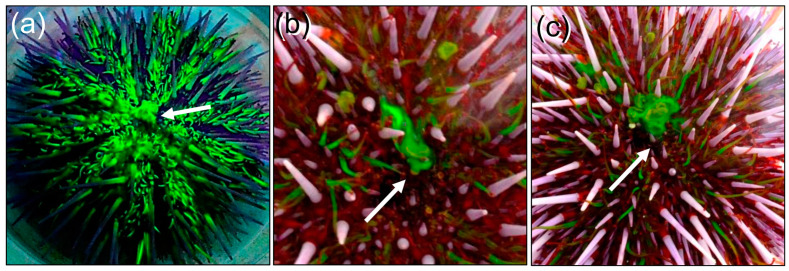
Fluorescein is excreted from the anus. (**a**) A sea urchin imaged under UV light shows fluorescein emerging from the anus (arrow) at 22 h 30 mpi. (**b**,**c**) A sea urchin imaged in sunlight at 23 hpi shows fluorescein emerging from the anus (arrows). The sea urchin in panel (**a**) is about 11 cm in diameter. These images were provided by Lee C. Heiman.

Injected sea urchins attached quickly with their tube feet to the side wall of the glass aquarium. Leakage of fluorescein from the injection site in the peristomial membrane was not observed, which was consistent with the effective clotting systems in echinoids [93,94]. The first evidence of visible green fluorescence in sea urchins under UV light occurred at 12 mpi, when three tube feet became fluorescent at the bottom side of an animal attached to the aquarium wall (Figure 7a). The fluorescence expanded to several nearby tube feet by 16 mpi (Figure 7b), to more tube feet by 23 mpi (Figure 7c), and to all of the tube feet by 30–90 mpi (Figure 7d). When sea urchins were observed in a culture dish at 50 to 120 mpi, the madreporite on the dorsal side was not fluorescent and the gills on the ventral side were not fluorescent; however, the ‘lips’ around the mouth and the buccal podia (Figure 6b) that surround the mouth were fluorescent (Figure 7e). Sea urchins were observed again at 21–23 hpi, when the tube feet remained fluorescent and the base of each spine was also fluorescent (Figure 7e, inset, arrows). Close inspection also showed that the edge of the anus was fluorescent (Figure 7f, inset, arrowhead), but the madreporite on the dorsal side (Figure 7f) and gills on the ventral side (Figure 7e) remained negative. As a sea urchin was righting itself after being inverted in a culture dish, fluorescein appeared in the water along the dorsal side of the animal (Figure 7g, arrows). Subsequent observations of a sea urchin at 22–23 hpi, both under UV light and in sunlight, showed that the source of the fluorescein in the water was excretion from the anus (Figure 8, arrows). Fluorescence in the tube feet slowly decreased over time, and by 10 days post-injection, it was no longer evident in the sea urchins. These observations indicated that when fluorescein was injected into the coelomic fluid of the perivisceral coelom, it appeared within minutes in the water vascular system, based on fluorescence of the tube feet. Furthermore, and in contrast to the predictions of excretion by the madreporite or the gills, excretion occurred by way of the intestine and anus.

## 4. Discussion

### The Haemal System as the Connection Between the Coelomic Fluid, the Water Vascular System, the Axial Organ, and the Intestine

The reduction in fluorescence in sea urchins over 10 days following fluorescein injection into the perivisceral coelom is consistent with metanephridial clearance by podocytes in the axial organ, and in accordance with glomerular filtering in vertebrates. The appearance of fluorescein in the tube feet within minutes after injection into the coelomic fluid indicates an open connection between the perivisceral coelom and the water vascular system. The flow of fluid between the axial complex and the stone canal of the water vascular system appears as a three-way connection at the aboral end of the axial complex that includes the haemal system [44,45,53]. Because the madreporite is part of the water vascular system and has a direct connection to the axial complex, its proposed function in excretion has been based on anatomy [15,51,52,53]. However, the failure to verify fluorescein excretion by the madreporite is consistent with sea water in-flow through the pores of the madreporite, which maintains hydraulic fluid pressure in the water vascular system of echinoderms, enabling movement of the tube feet [95,96,97]. The gills are connected with the fluid in the peripharyngeal coelom that encloses Aristotle’s Lantern [40] and they have been reported to function in excretion [92]. Their failure to show fluorescence, as observed for the tube feet, or to excrete fluorescein, suggests that the gills are not involved in excretion, or that the peripharyngeal coelom does not have an open connection to the perivisceral coelom, the water vascular system, or the haemal system.

The elimination of fluorescein from the anal opening is similar to the appearance of red fluorescent fluid eliminated from the anus of the green sea urchin, *Strongylocentrotus droebachiensis* [98]. Although a fluid exudate from the anus of sea urchins has not been considered previously, it suggests the possibility that fluorescein and other waste molecules may be transported directly from the coelomic fluid into the intestinal lumen by cells in the gut wall. There are a number of genes that encode proteins involved in ammonia transport in the intestines of non-nephrozoans [12], of which several are expressed in the sea urchin intestine and the axial organ, including an ammonia transporter, an H^+^-ATPase, and several aquaporins (Table 3). In addition, an alternate route for excretion may be the transport of waste material contained in the phagosomes of phagocytes that migrate from the axial organ through the rectal wall and into the lumen [99]. However, because echinoderms are members of the nephrozoan clade, the direct transport of waste into the intestinal lumen from the coelomic fluid may not be a major excretory mechanism. Consequently, this begs the question of how fluorescein—and, by inference, metabolic waste in general—in the filtrate from the podocytes in the axial organ is connected to and enters the intestine. A key aspect of the observation reported here may be the delayed appearance of fluorescein from the anus relative to its appearance in the tube feet, which occurs within minutes of injection into the coelomic fluid. The basis for this delay may be informative for understanding the excretion mechanism, which may not be consistent with fluorescein delivery by phagocytes to the intestine, or by intestinal transporters. Although the location of the axial organ in the adult is in close association with both the esophagus and the rectal tissues (Figure 5a) [51,53], there is no physical or open connection between the axial complex and the esophagus or rectal walls that is observable or has been documented based on anatomy or histology [16,51,53]. The fluid in the water vascular system is also not connected directly to the intestine. Consequently, the haemal system may be a key to the excretory process.

The ventral (oral) end of the axial complex is connected to the oral haemal ring located on the top of Aristotle’s Lantern, and runs in parallel to the ring canal of the water vascular system (Figure 5b) [53]. A branch of the haemal system called the ventral, inner, or intestinal haemal vessel (also called the inner marginal sinus) originates from the oral haemal ring and runs along the external surface of the esophagus to the inner (adaxial) edge of the first loop of the intestine that is positioned in the ventral or oral region of the sea urchin (Figure 5c) [45]. At the intersection of the esophagus and the intestine, the ventral haemal vessel branches into a network of capillaries in the intestinal wall (Figure 5c) [53,55,100]. The dorsal (aboral) end of the axial complex is located at the intersection of the stone canal that is part of the water vascular system and the dorsal haemal vessel that arises directly from the axial complex. The dorsal haemal vessel connects to the second or upper loop of the intestine in the dorsal region of the sea urchin (Figure 5b) and haemal capillaries branch into the intestinal wall (Figure 5c) [53,55]. Both of the haemal vessels and their branched capillaries connect the axial complex directly to the intestine.

Our findings suggest a different route for echinoid excretion compared to anatomical predictions [45,51,53], which involves the axial organ and the intestine, leading to a testable hypothesis. Fluorescein in the coelomic fluid of the perivisceral coelom enters the water vascular system and moves to the axial complex at the intersection with the stone canal. The fluid undergoes filtration by the podocytes in the axial organ, driven by pressure from the contractile function of the pulsating vessel of the axial organ and moving fluid to the haemal system [45]. The haemal vessels transport the filtrate to the intestine, where it is transferred to the intestinal lumen by the capillaries. Transfer through the walls of the haemal capillaries and the intestine may occur by molecular transport mechanisms, as suggested by the expression of genes encoding ammonia transporters (Table 3). Although it has been speculated that the haemal system surrounding the intestine functions in nutrient absorption, and that the fluid in the haemal capillaries flows from the intestine into the haemal system [101], this concept is based on an assumed similarity to the closed circulatory system in vertebrates and the transport of nutrients from the intestine to the liver by the portal vein. The haemal system in echinoderms is not a closed circulatory system; the capillaries end blindly in the intestinal wall. Rather than directional flow, alternating flow of fluid in the haemal vessels associated with the intestine has been reported [101]. This suggests that waste removal may occur in this system when fluid flow is directed toward the intestine. Overall, the haemal system may function to transfer filtered fluid with waste material, in this case including fluorescein, from the axial complex through the haemal vessels and capillaries to the intestinal wall, and into the lumen for excretion [44].

## 5. Conclusions and Future Directions

The axial organ is the only relatively large organ in echinoids aside from the intestine and the gonads. Its function and importance in echinoderms has confused researchers since the 1800s and has resulted in ongoing debates. It is feasible that it carries out multiple functions that may have underpinned the confusions about this organ in echinoderms. Here, the axial organ in adult sea urchins is proposed to have a metanephridial function, and it acts as a key site for the clearance of fluorescein, and by inference, metabolic waste products that are transferred to the intestine by the haemal system. However, this hypothesis requires further experimental verification with live adult sea urchins. Collecting and evaluating rectal fluid to detect ammonia and other waste products might be a first step in future analysis. The next step would be to observe the movement of fluorescein in the fluid of the water vascular system, the axial complex, the haemal system, and the intestinal lumen, but this is challenging because adult sea urchins are not transparent. The internal calcareous skeleton, or test, that surrounds the adult body makes surgery and observations of live internal anatomy impossible. Dissecting sea urchins after fluorescein injection to evaluate key structures and connections among the fluid-filled vessels and spaces is challenging because any dissection destroys at least some of the internal anatomy. An alternative approach to test the hypothesis of the metanephridial function and fluid filtering by podocyte cells in echinoids would be to inject fluorescein into the blastocoel of clear sea urchin larvae. This would enable visualization of fluorescein clearance based on filtration by podocytes that line the left coelom, followed by fluorescein accumulation in the lumen, flow through the water or pore canal to the hydropore, and the appearance of fluorescein on the dorsal exterior of the larvae. Tracking fluorescein flow may also be observed in small adult sea urchins injected with fluorescein by incubating live animals in sea water with tartrazine, a water soluble dye that renders tissues transparent [102]. Alternatively, the transparency of tissues including the test, can be accomplished for injected sea urchins that are fixed and decalcified in a hydrogel of 30% acrylamide called See-Star that maintains the structural integrity of small marine invertebrates [103]. Animals cleared with Sea-Star could be used to visualize the location of fluorescein during filtration and excretion at a series of time points after injection into the perivisceral coelom. Outcomes from these proposed research approaches may establish the location and functions of the excretory system in echinoderms, including filtration by the metanephridial activities of the axial organ and the route by which waste material enters the intestine. The results are expected to establish the presence of an echinoid excretory system and lay the groundwork for future research to characterize this system for all classes of echinoderms.

## Figures and Tables

**Table 1:** Podocyte-specific genes in the genome of the purple sea urchin, *Strongylocentrotus purpuratus*, are expressed in the axial organ.

Vertebrate Protein	Function	Human Accession Number (GenBank, NCBI.gov)	Sea Urchin Protein Match ^1^	Sea Urchin Gene Match, e Value	Genome Location; LOC Number ^2^ RNAseq; WHL22 Number ^3^	Tissue of Elevated Gene Expression Post-Metamorphosis ^3^
Human nephrin	slit diaphragm structure	AAG17141.1	Nephrin	8 × 10^−122^	LOC592278 WHL22.293015.0	Axial organ
			Nephrin-like	Gene name search	LOC581043 WHL22.22857	Axial organ, coelomocytes
Mouse NEPH1	nephrin/podocin-interacting protein	AAN73043.1	Kin of IRRE-like protein ^4^	1 × 10^−80^	LOC100890311 WHL22.526524.1	Axial organ elevated relative to other adult tissues
Human Tie2	receptor tyrosine kinase, angio-poietin receptor	KAI4006839.1	Tie1/2 ^5^	6 × 10^−109^	LOC110977718 or LOCtek WHL22.41577	Coelomocytes and axial organ
Human SIX1	Homeobox transcription factor	CAA62974.1	Six1/2 ^6^	2 × 10^−130^	LOC110974175 WHL22.121485	Axial organ, coelomocytes, testes, and radial nerve
Human SIX2	Homeobox transcription factor	NP_058628.3	Six1/2 ^6^	2 × 10^−121^		

^1^ BLASTp matches to sea urchin proteins deduced from the gene sequences; genome (ver 5.0). ^2^ Protein matches are based on BLASTp searches of echinoderm proteins on Echinobase.org. ^3^ RNAseq results are from Tu et al. [60] and available for Echinobase.org. ^4^ Kin of IRRE is another name for NEPH1 of the nephrin family adhesion molecules. ^5^ Expression in the axial organ and coelomocytes reported by Stevens et al. [81] is verified by the RNAseq data set of Tu et al. [60]. ^6^ Six1/2 is the sea urchin orthologue of both six1 and six2 proteins in humans.

**Table 2:** Podocyte-specific and glomerular-specific gene expression are identified in the transcriptome of the nurse shark, *Ginglymostoma cirratum*.

Vertebrate Protein	Query Genbank Accession Number	e Value of Top tBlastn Hit ^1^
Mouse NEPH1	AAN73043.1	0
Human Nephrin	AAG17141.1	0
Danio podocin	NP_001018155.2	8 × 10^−61^
Human Tie2	KAI4006839.1	0
Human six1	CAA62974.1	Not found ^2^
Human six2	NP_058628.3	Not found ^2^

^1^ Nurse shark podocyte marker orthologues were identified by tBlastn searches against the nurse shark transcriptome (GenBank BioProject number PRJNA841433) using the corresponding vertebrate protein sequences. ^2^ Orthologues for the podocyte markers six1 and six2 were not identified in the nurse shark transcriptome.

**Table 3:** Some ammonia transport genes are expressed in the sea urchin intestine.

Sea Urchin Gene	Genome Location Number ^1^	Transcript Number ^2^	Elevated Gene Expression Post-Metamorphosis
*Ammonium transporter 1*	LOC576563	WHL22.591474	Intestine
*V-type H^+^-ATPase, type A*	LOC585744	WHL22.470441	Intestine, axial organ coelomocytes, nerve, juvenile
*Aquaporin 12A-like*	LOC115918744	WHL22.456167	Intestine
*Aquaporin*	LOC105444576	WHL22.273709	Axial organ
*Aquaporin 3*	LOC105442651	WHL22.528654	Axial organ, intestine, testes, nerve, juvenile
*Aquaporin 8*	LOC589852 LOC762564	WHL22.362925 WHL22.318239	Intestine, axial organ

^1^ Location numbers for genes in the *S. purpuratus* genome (ver 5.0). ^2^ The sea urchin RNAseq data set from Tu et al. [60] is available from the genome browser at www.Echinobase.org.

## Data Availability

All data associated with this study are presented in this paper.

## Abbreviations

The following abbreviations are used in this manuscript:

GBM	Glomerular basement membrane
TPM	Transcript per million
mpi	Minutes post-injection
hpi	Hours post-injections
NEPH1	Nephrin/podocin-interacting protein 1
TEM	Transmission electron micrograph
UV	Ultraviolet
